# Bio-Nanocomposites Obtained by Green Synthesis with Silver Nanoparticles from By-Products of Fruits, Berries and Vegetables: Structure, Functionality and Applications—A Review

**DOI:** 10.3390/nano16181169

**Published:** 2026-09-16

**Authors:** Vaidė Sakalauskienė, Syeda Hijab Zehra, Aiste Balciunaitiene, Jonas Viskelis

**Affiliations:** Institute of Horticulture, Lithuanian Research Centre for Agriculture and Forestry, 54333 Babtai, Lithuania

**Keywords:** green synthesis, by-products of production, silver nanoparticles (AgNPs), phytochemical analysis, antibacterial activity, antioxidant activity

## Abstract

Silver nanoparticles (AgNPs) are widely studied as functional nanomaterials due to their exceptional antimicrobial properties, allowing their application not only in food packaging but also in medicine, cosmetics, and other biomedical and industrial fields. In recent years, special attention has been paid to “green” synthesis methods, in which AgNPs are obtained using by-products of fruit, berry, and vegetable production, such as peels, pomace, and seed waste. These bioactive extracts act as natural reducing and stabilizing agents, allowing the production of more environmentally friendly, less toxic nanoparticles while contributing to reducing the waste of limited resources and implementing the principles of the circular economy. AgNPs synthesized in this way are successfully integrated into biopolymeric matrices (e.g., chitosan, starch, alginates), forming functional nanocomposite films and coatings. These systems exhibit improved mechanical and barrier properties, increased thermal stability, and enhanced antimicrobial activity. The biopolymeric matrix also allows for the control of silver ion release, reducing potential cytotoxic effects and enhancing safety in various applications. In summary, these sustainable nanocomposite systems are promising in various fields, including food technology, medicine, and cosmetics, contributing to more efficient resource utilization and environmental protection.

## 1. Introduction

Reducing food waste is one of the prominent goals in current research, a target which has been set by the United Nations to achieve a more sustainable world by 2030 [1]. Food losses pose a significant threat to both global economic policy and food supply security [2]. These food wastes are part of unintentional and intentional losses, which lead to the wastage of agricultural by-products [1]. While some sources argue that losses primarily occur during post-harvest stages, others emphasize the role of supply chain inefficiencies throughout the entire production and distribution process [2]. More than a billion tons of food are discarded worldwide each year—approximately one-third of the total amount produced. In other words, approximately 1.3 billion tons of food are discarded annually, which would have required 1.4 billion hectares of fertile land for production, accounting for nearly one-third of the world’s arable land [3]. Waste from fruits and vegetables makes up approximately 16% of the overall food waste and contributes around 6% to global greenhouse gas emissions. The term “fruit and vegetable waste” (FVW) refers to all waste generated at different stages of the supply chain, starting from the farmers’ harvest and ending with unsold food in retail, public catering establishments, and the final consumer, in addition to the processing, preservation, and packaging processes [4]. This waste can be further processed and variously recovered or at least preserved without loss. By-products generated during industrial processing—inedible components such as peels, seeds, pomace, or stems—are also classified as FVW. FVW is characterized by valuable properties such as biological degradability, economic efficiency, durability, and their light weight, which give them great potential for further use [5,6].

Perhaps an even bigger global problem is plastics and their products. Plastics, due to their properties such as flexibility and durability, have become a revolution in industry and everyday life since 1950, with production increasing from two million tons to 380 million tons between 1950 and 2015. It is estimated that by 2030, global plastic production will reach 550 million tons [7,8]. One of the major disadvantages of plastic is that most of it is single-use, including disposable products and packaging [7,9]. Many food products are transported in plastic packaging, which, as established in empirical studies, constitutes a significant source of plastic dispersion in the environment [10]. Additionally, plastic materials are widely used in various sectors, including households, agriculture, medicine, the pharmaceutical industry, engineering, and scientific research. Considering the extent of plastic usage and the environmental impact of its decomposition products—particularly microplastics—it is essential to bring more attention to the issue of microplastic pollution in everyday human activities [11,12].

The increasing consumer demand for fresh, longer-lasting, and quality-controlled products encourages manufacturers to implement advanced, safe packaging systems. This poses significant challenges to the food packaging sector and simultaneously acts as a stimulus for the creation of new, technologically advanced packaging concepts. One way to meet these increased expectations is to develop antimicrobial packaging that reduces food product losses due to extended shelf life and protects consumers from diseases caused by food spoilage [13,14,15]. To ensure the freshness of packaged food products, such as fresh fruits, vegetables, dairy, or meat products, various packaging solutions are applied. Packaging systems, such as films, bags, or absorbent layers, can be integrated with antimicrobial agents, antioxidants, carbon dioxide absorbers, and other components. They release or absorb active chemical substances that affect the product or the medium around it, thus extending food stability and improving the overall functionality of the packaging [15]. Antimicrobial substances embedded in food packaging effectively limit the proliferation of pathogenic and spoilage-causing microorganisms, thereby contributing to a longer storage period for products. Therefore, these substances are one of the most commonly applied types of active components in commercial food storage systems. As a result, advanced packaging solutions that more effectively protect products are being developed, such as active and smart packaging, modified atmosphere systems, and the application of nanotechnology in packaging materials [13,16].

### Strategy of Literature Search and Methodology

To ensure representative, transparent and comprehensive data, a literature review has been conducted on green synthesis of silver nanoparticles (AgNPs), bio-nanocomposites, food valorization and food by-products, and a strategic structured literature search has been carried out with high effort.

A deep search has been conducted through all available electronic databases, consisting of Scopus, PubMed, paid journals, ScienceDirect, Google Scholar and Web of Science; data published between 2010 and 2026 was selected. Special keywords were used while searching published articles such as “fruits and vegetable residue”, “bio-nanocomposite”, “active food packaging”, “chitosan”, “silver nanoparticles”, “AgNPs”, “berry pomace”, “antioxidant activity”, “antimicrobial activity” and “food valorisation” by using suitable Boolean operators.

## 2. Metal Nanoparticles and Silver Nanoparticles

### 2.1. Mechanism of Green Synthesis of Silver Nanoparticles

Among various nanomaterials, such as metals (Ag, Au, Cu, etc.), metal oxides (ZnO, TiO_2_, etc.), and non-metallic oxides (SiO_2_), silver nanoparticles stand out due to their unique properties and effective biological impact. Humanity has been using silver for about 7000 years, and science recognizes it as a very strong antibacterial agent capable of destroying numerous microorganisms that cause various infectious diseases. Silver nanoparticles are widely applied in packaging systems. However, traditional nanoparticle production methods based on chemical and physical processes raise concerns about potential negative impacts on health and the environment. For this reason, increasing attention is being paid to sustainable solutions, particularly green nanoparticle synthesis using plants or their biologically active compounds. Clinical application of ionic silver is limited due to its pronounced toxicity and various adverse effects, such as argyria, allergic reactions, leukopenia, toxic effects on bone marrow, and potential kidney or liver damage. It has been established that when silver ions are converted into metallic silver nanoparticles (AgNP) using biological and other alternative synthesis methods, their toxicity is significantly reduced, while antimicrobial activity is notably increased. Due to these properties, AgNP are considered a promising tool for the clinical treatment of infectious diseases [17,18,19]. Commonly used nanoparticles include metallic, metal oxide, carbon-based nanomaterials, and quantum dots. As the dimensions of nanoparticles decrease, their surface-to-volume ratio increases, allowing more atoms or molecules to fit within the same volume unit. Consequently, a smaller amount of material is sufficient to achieve the desired activity or specific properties [20].

Although the antibacterial activity of silver nanomaterials is widely recognized and extensively described in the scientific literature, the question of how nanoparticle size and morphology determine their antimicrobial effectiveness remains a subject of debate. Many studies emphasize that anisotropic shapes of silver nanoparticles, such as nanoplates or triangular nanoprisms, can have a significant impact on enhanced biocidal activity. For example, some works have found that truncated triangular AgNPs exhibit greater antibacterial effectiveness compared to spherical or rod-shaped particles. Other authors have also reported that triangular silver nanoprisms with sharp edges and pointed tips demonstrate a more pronounced antiseptic effect than spherical or nearly spherical nanoparticles. This increased activity is often associated with specific crystallographic properties, especially basal planes and high atom density edges, which act as centers of high chemical reactivity, thereby enhancing the antibacterial effect. On the other hand, some scientific publications indicate that isotropic silver nanoparticles, especially in spherical form, can exhibit high antibacterial efficacy. In this case, the main argument is based on the high surface-to-volume ratio of spherical nanoparticles, which ensures a large interaction area with microorganisms and results in high reactivity and antimicrobial activity [21]. Based on experimental and theoretical data, nanoparticles with dimensions in the range of 1–100 nm are considered optimal for practical application [22].

### 2.2. Characterization of Silver Nanoparticles

Various analytical methods are used to investigate the physicochemical properties of nanoparticles, allowing for the assessment of their morphology, structure, particle size distribution, surface area, and optical characteristics [23].

#### 2.2.1. Morphology SEM, TEM,

**Figure 1:**
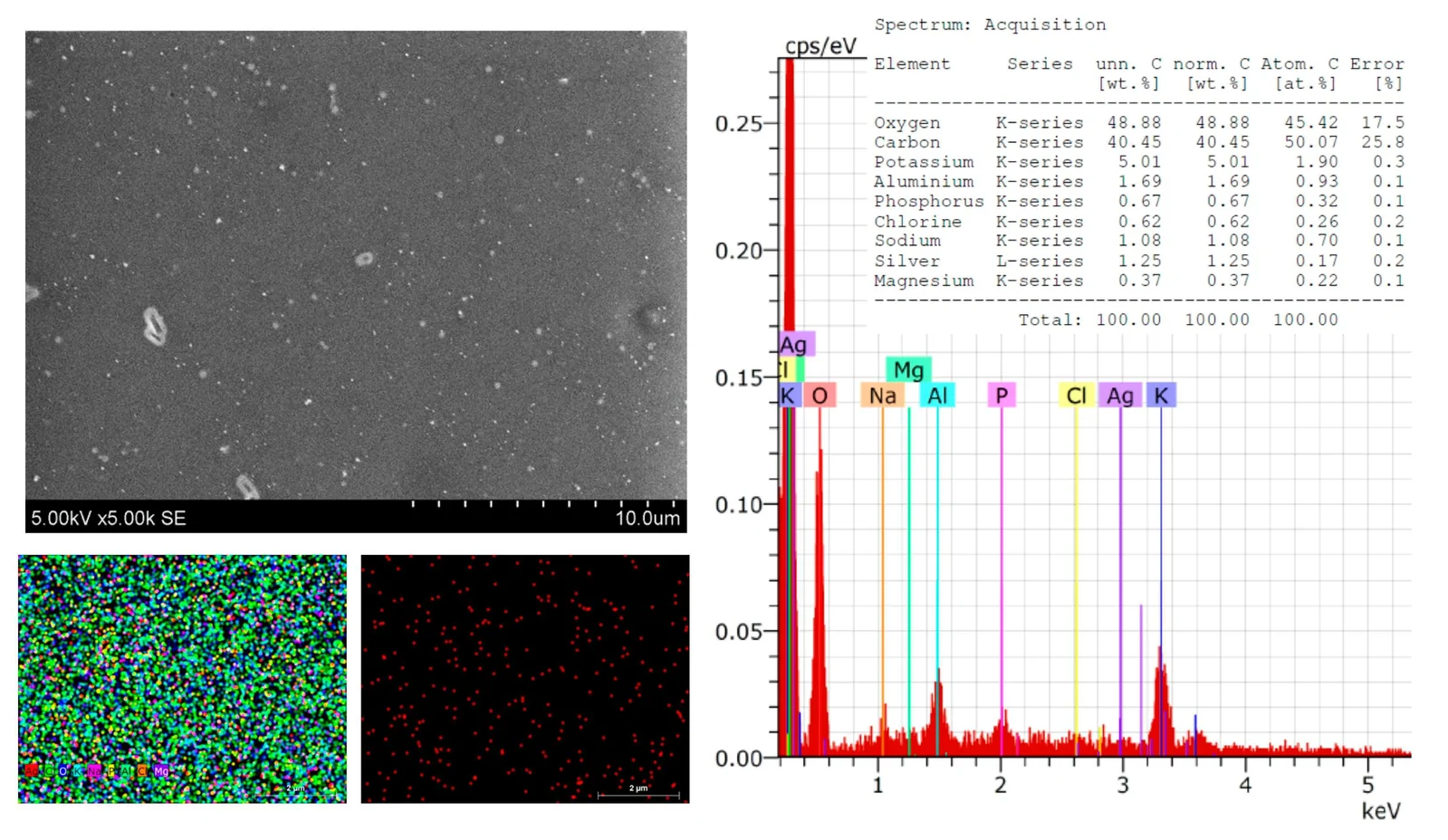
SEM images and EDS spectra and element mapping of *Sym. Radix*/AgNPs, adapted from [25].

The scanning electron microscope (SEM) is one of the most versatile instrumental methods, allowing for a detailed examination of the morphology of microstructural formations and their chemical composition characteristics. SEM is used to obtain high-resolution images and perform detailed analysis of sample surfaces. An example is shown in Figure 1. It is an electron microscopy method where a focused electron beam is used to scan the sample surface, and the obtained images have significantly higher resolution than those from optical microscopy. Depending on the device configuration, the SEM resolution can range from less than 1 nm to several nanometers [24].

In addition to the visual information provided by the SEM images, EDX analysis confirmed the elemental composition of the nanoparticles [26].

**Figure 2:**
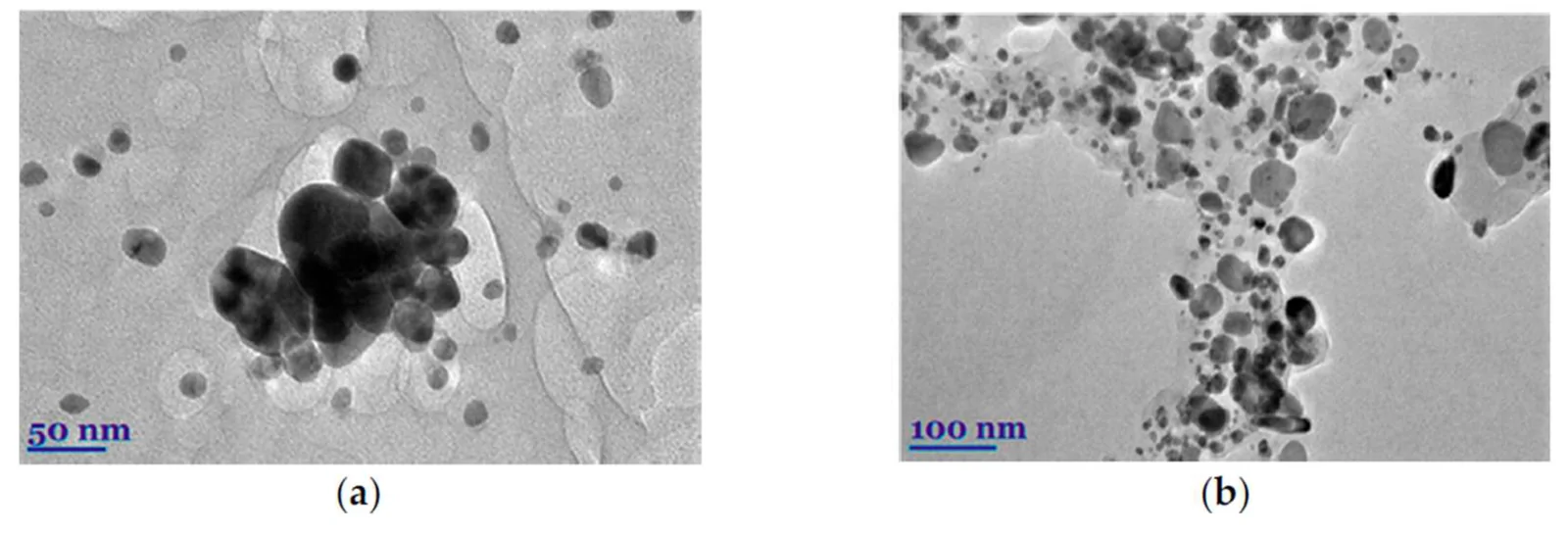
Images of biosynthesized Sym. Radix/AgNPs-3 (**a**,**b**) in different magnifications, adapted from [25].

Transmission electron microscopy (TEM) is a high-resolution structural analysis method widely applied in nanoscience, materials science, and life sciences research. This technique is based on the interaction of a high-energy electron beam with ultra-thin samples, allowing information about their internal structure at the atomic scale to be obtained. Unlike optical microscopy, which is limited by the diffraction limit of the visible light wavelength, TEM has exceptionally high spatial resolution and allows magnifications up to 50 × 10^6^ times. In Figure 2, we see an image magnified 50 and 100 times. These capabilities are ensured by the extremely short wavelength of electrons accelerated in a strong electromagnetic field, which is approximately five orders of magnitude shorter than visible light, making detailed imaging of nanometric and atomic structures possible [17,27,28,29].

#### 2.2.2. Structural Analysis (UV-Vis, F-TIR)

**Figure 3:**
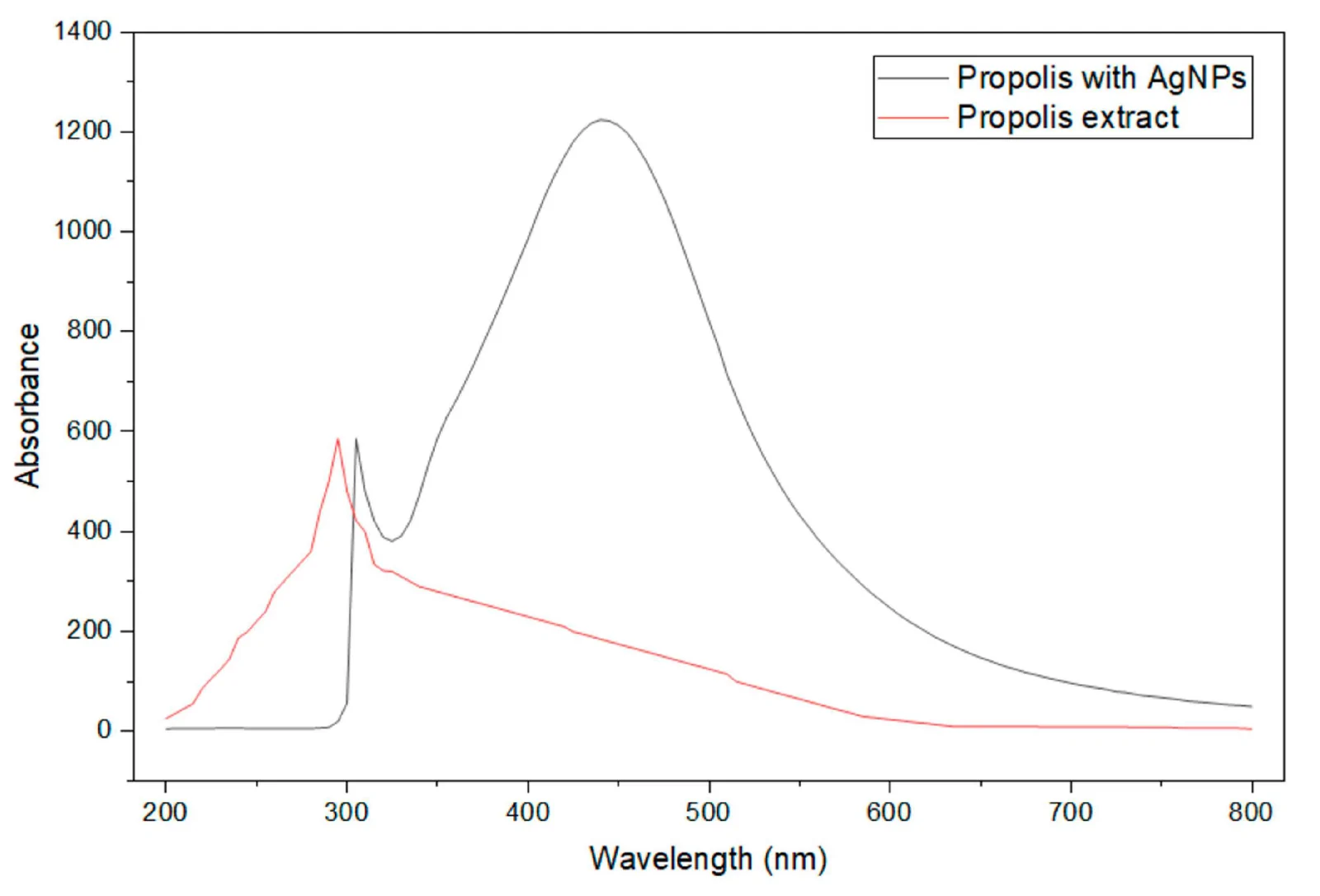
UV-Visible spectra of propolis and propolis with silver nanoparticles, adapted from [28].

UV-visible spectroscopy is a simple and widely applied analytical method used to monitor the formation process of silver nanoparticles (AgNPs). The free conduction electrons present in metallic nanoparticles can collectively oscillate in resonance mode within certain wavelength intervals when interacting with an external electromagnetic field, causing the surface plasmon resonance (SPR) phenomenon. SPR excitation results in the characteristic color of AgNP colloidal solutions and specific absorption bands in the UV-Vis spectrum. The formation of silver nanoparticles is usually confirmed by observing the SPR absorption maximum around 435 nm, which is associated with the reduction of silver ions to metallic AgNP. Typically, spherical nanoparticles exhibit a single distinct SPR absorption band, whereas anisotropic particles are characterized by two or more SPR bands, whose position and intensity depend on the morphology of the particles. Furthermore, the absence of absorption maxima around the wavelength regions of approximately 335 and 560 nm is often interpreted as an indicator of the absence of nanoparticle aggregation [30]. Figure 3 shows the UV-visible spectra of Propolis and Propolis with silver nanoparticles.

Fourier transform infrared spectroscopy (FTIR) is a widely applied analytical methodology used to identify functional groups on the surface of investigated nanoparticles. FTIR analysis allows the determination of the interaction of bioactive molecules present in plant extracts with the surface of silver nanoparticles (AgNPs), which are responsible for the stabilization of nanoparticles and the formation of coating layers. Spectral data provide information about possible chemical interactions, such as reduction, coordination, or hydrogen bonding between AgNP and polyphenols, flavonoids, or other biomolecules present in the extract. The results of FTIR studies are often used in conjunction with TEM and UV–Vis spectroscopy to thoroughly characterize the structure of nanoparticles, their surface chemical composition, and stability in solution. 

FTIR spectroscopy is commonly used to identify functional groups involved in the reduction and stabilization of AgNPs synthesized using plant extracts. Marukurti et al. [31] reported characteristic FTIR absorption bands for AgNPs synthesized using the methanolic leaf extract of Plumeria rubra. The major vibrational bands observed at approximately 1374 and 1588 cm^−1^ were attributed to functional groups associated with phytochemical constituents present in the plant extract. Additional bands across the investigated spectral region indicated the presence of organic compounds on the nanoparticle surface, suggesting their involvement in the reduction of Ag^+^ ions and subsequent stabilization of the formed AgNPs [31].

#### 2.2.3. Physical Properties (Color Indexes, TA.XTPlus Texture Analyser, Dzeta-Potentiometer Zeta Sizer Nano Z)

Silver nanoparticles (AgNPs) exhibit exceptional physical properties, including the optically visible color of colloidal solutions, which is directly related to surface plasmon resonance (SPR). The SPR phenomenon occurs due to collective oscillations of free electrons on the surface of nanoparticles interacting with electromagnetic radiation. The resonant frequency of these electronic oscillations depends on the size, shape, surface chemical composition of the nanoparticles, and the dielectric constant of the environment.

Color changes in AgNP colloids are often quantified using color indices such as:

- The CIE L*a*b* system describes the lightness (L*), red/green (a*), and blue/yellow (b*) components;
- RGB values—based on electronic images or spectrophotometric measurements;
- Absorption maximum in the UV-Vis spectrum—used as a quantitative SPR indicator, typically ~430–440 nm for spherical AgNPs [32,33].

The analysis of these color indices allows monitoring the distribution of nanoparticle sizes, changes in morphology, and aggregation processes, and it is practically used for observing AgNP synthesis and optimizing control parameters. The table below shows how AgNP size, morphology, and color indices are related through the UV–Vis SPR absorption maximum and color parameters. The relationship between AgNP size and morphology, the position of the UV–Vis surface plasmon resonance (SPR) absorption maximum, and the corresponding color characteristics of colloidal AgNP solutions is summarized in Table 1.

Key Points in the Scheme:

- The SPR absorption maximum (λ_max_) for silver nanoparticles typically lies between ~400 and 500 nm and shifts toward longer wavelengths (red shift) as particle size increases or shape becomes anisotropic.
- These SPR shifts underlie the observable color changes in colloidal AgNP solutions (e.g., from yellow to brown) [26].
- Color indices such as RGB or CIE L*a*b* can be quantitatively correlated with such SPR changes for more precise optical analysis (e.g., using colorimetric or spectrophotometric measurements). In 1976, the CIE L*a*b* color space was defined according to the tristimulus values L*, a*, and b*. This space has a cubic structure, where the L* axis is oriented vertically, from top to bottom. The L* value describes lightness: the highest value of 100 corresponds to a fully diffusing white surface, while the lowest value of 0 represents absolute black. The a* and b* axes do not have strict numerical limits. A positive a* value corresponds to red, while a negative value corresponds to green; a positive b* value corresponds to yellow, while a negative b* corresponds to blue. This system allows for the quantitative description of color independently of lighting conditions, and is widely used in optical and colloidal systems, including the study of color in nanoparticle dispersions [34].

**Figure 4:**
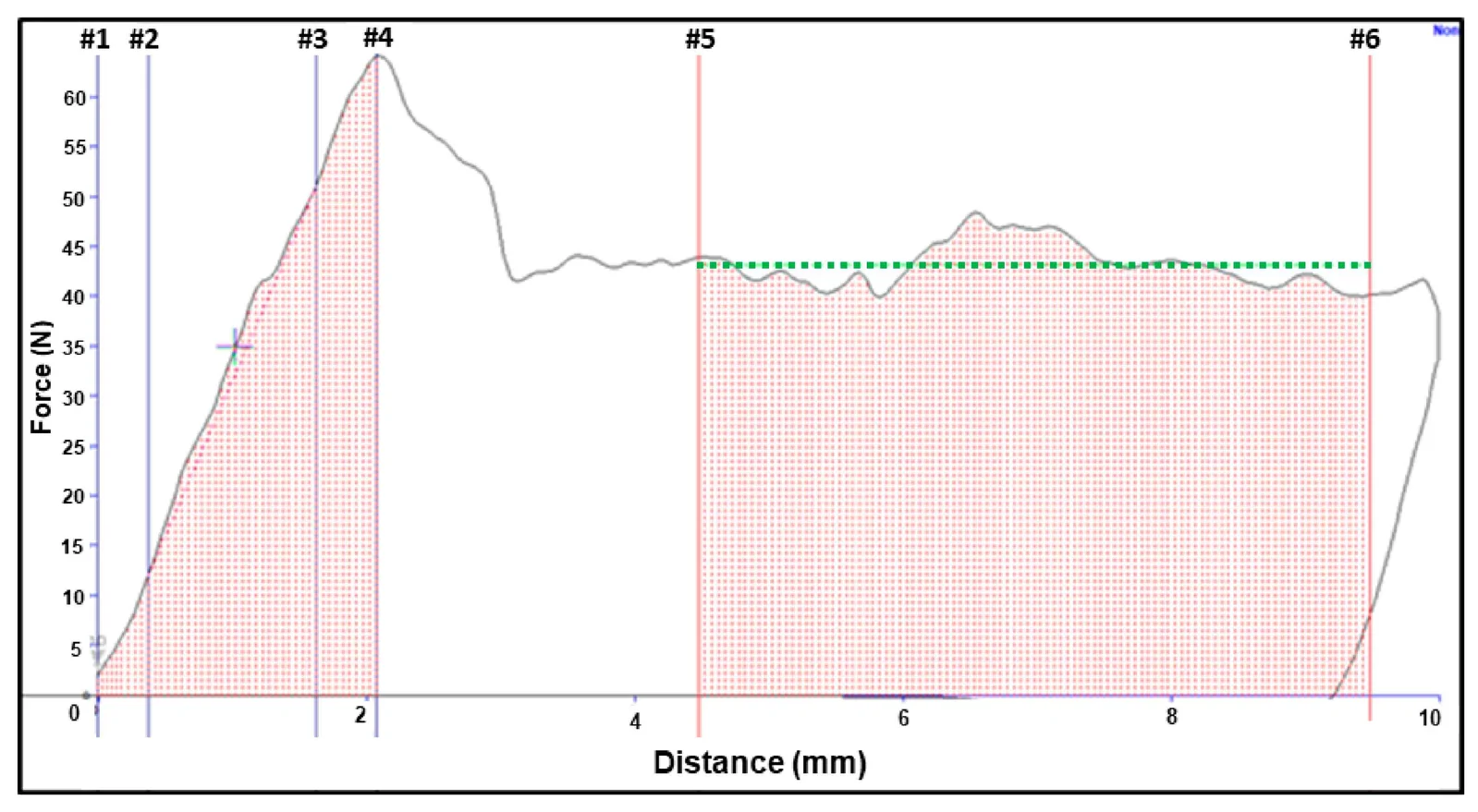
Example of force-distance curve obtained from TA.XT*plus* Texture Analyzer (Stable Micro Systems Ltd., Godalming, Surrey, UK) resulting from penetration of an unpeeled apple. The TA.XT*plus* was equipped with an 8 mm cylindrical stainless-steel probe and operated to a depth of 10 mm at a speed of 10 mm/s, adapted from [35].

TA.XT*plus* is a texture analyzer that can quantitatively determine the mechanical properties of materials, such as hardness, fracturability, adhesiveness, gel strength, elasticity, and other textural parameters. The device operates by applying a controlled force or deformation to a sample using a moving arm (circular clamp), and the force and distance data are recorded as a distorted curve from which textural parameters are calculated. The instrument features robust column design load cells, which allow testing from very small forces up to ~50 kg or more (depending on the model), and is programmable with various scenarios, such as compression, penetration, or tension tests. Application areas are (a) food product research: texture control, formula optimization, product quality assurance; (b) cosmetics and pharmacy: analysis of the mechanical properties of creams, gels, tablets, and other substances; (c) materials science: study of mechanical properties for polymers, foams, or composites [35]. A representative force–distance curve used to evaluate the tensile behavior of the biopolymer film, including its maximum force and elongation at break, is presented in Figure 4.

The ZetaSizer Nano Z/Nano ZS is a high-precision analytical device designed to study the physical properties of nanometer-sized particles and colloidal systems in liquid media. Using laser-optics-based methods, this analyzer allows the determination of the hydrodynamic size and size distribution of particles by applying the dynamic light scattering (DLS) principle, as well as evaluating the electrokinetic surface properties expressed as zeta potential, which is calculated based on the electrophoretic mobility of particles measured by electrophoretic light scattering (ELS). Depending on the configuration, the device can also be used for determining molecular mass and other colloidal parameters.

Preparation, purification, and functional property analysis of nanoparticle systems largely rely on the control of particle size and surface characteristics, as these factors directly determine colloid stability, interaction with the environment, and potential application. In both inorganic and organic or soft colloids, particle surface charge, chemical composition, and zeta potential are essential parameters that shape their structural stability and functional activity. Such systems can be purposefully modified to enhance their biological compatibility, ensure selective interaction with cell membranes, or develop desired optical and reactive properties.

Dynamic light scattering (DLS) is a widely used technique for evaluating the hydrodynamic size of nanoparticles and their aggregation state in dispersion [36,37]. The method is based on the analysis of fluctuations in scattered light intensity arising from the Brownian motion of particles. The translational diffusion coefficient (D) is derived from the autocorrelation function of these intensity fluctuations and is related to the hydrodynamic size of the particles [36]. 

The hydrodynamic particle diameter is calculated using the Stokes–Einstein equation [38,39]:

$$d_{h}=\frac{k_{B}T}{3\pi \eta D}$$

where $d_{h}$ is the hydrodynamic particle diameter, $k_{B}$ is the Boltzmann constant, T is the absolute temperature, η is the dynamic viscosity of the dispersion medium, and D is the translational diffusion coefficient. The size determined by DLS represents the hydrodynamic rather than the direct geometric diameter of the particle and may therefore differ from particle sizes determined by electron microscopy. Moreover, DLS is particularly sensitive to larger particles and aggregates, which can contribute disproportionately to the scattered light signal [36,37].

Zeta potential (ζ) is an electrokinetic parameter used to evaluate the surface properties and colloidal stability of nanoparticle dispersions. Its value depends not only on the properties of the particles themselves but also on the composition of the dispersion medium and other measurement conditions [36,38]. Zeta potential is determined from the experimentally measured electrophoretic mobility ($\mu_{e}$) of the particles, and the relationship between these parameters can be described by Henry’s equation [36,38]:

$$\mu_{e}=\frac{2\varepsilon_{r}\varepsilon_{0}\zeta f\left(\kappa a\right)}{3\eta }$$

or, when expressed in terms of zeta potential:

$$\zeta =\frac{3\eta \mu_{e}}{2\varepsilon_{r}\varepsilon_{0}f\left(\kappa a\right)}$$

where $\mu_{e}$ is the electrophoretic mobility, η is the dynamic viscosity of the dispersion medium, $\varepsilon_{r}$ is the relative permittivity of the medium, $\varepsilon_{0}$ is the vacuum permittivity, a is the particle radius, κ is the inverse electrostatic screening length of the electrical double layer, and $f\left(\kappa a\right)$ is Henry’s function [38]. In the Hückel approximation, $f\left(\kappa a\right)=1$, whereas in the Smoluchowski approximation, $f\left(\kappa a\right)=1.5.$ The Smoluchowski approximation is applicable when the electrical double layer is much thinner than the particle radius, whereas the Hückel approximation applies when the electrical double layer is much thicker than the particle radius [38].

During DLS measurements, the scattered radiation from particles undergoing Brownian motion is detected as a function of time. The translational diffusion coefficient D is obtained from the autocorrelation of the detected intensity, and the hydrodynamic diameter $d_{h}$ is subsequently calculated using the Stokes–Einstein equation [37]. The temperature T represents the measurement temperature, η is the dynamic viscosity of the fluid in which the particles are dispersed, and $k_{B}$ is the Boltzmann constant [37]. During zeta potential measurements, particles exposed to an external electric field move with a velocity and direction that depend on the applied field and particle properties. Their electrophoretic mobility $\mu_{e}$ can be determined using Phase Analysis Light Scattering (PALS), and the zeta potential ζ is subsequently estimated from the measured electrophoretic mobility using an appropriate electrokinetic model [38]. The relative permittivity $\varepsilon_{r}$ characterizes the dispersion medium, whereas $\varepsilon_{0}$ is the permittivity of vacuum. The inverse Debye screening length κ describes the electrical double layer, while a represents the particle radius; together, these quantities define the parameter κa used in Henry’s function $f\left(\kappa a\right)$. The Hückel and Smoluchowski approximations correspond to $f\left(\kappa a\right)=1$ and $f\left(\kappa a\right)=1.5$, respectively, depending on the relationship between the electrical double-layer thickness and particle radius [38].

Thus, in DLS, the translational diffusion coefficient (D) is obtained from fluctuations in scattered light intensity, and the hydrodynamic diameter ($d_{h}$) is subsequently calculated using the Stokes–Einstein equation. In zeta potential analysis, the electrophoretic mobility ($\mu_{e}$) is experimentally determined, and ζ is then calculated using an appropriate electrokinetic model [37,38].

Zeta potential is considered one of the most important criteria characterizing the stability of colloidal dispersions, as it reflects the electrostatic repulsion forces between particles in suspension. Additionally, the surface charge of particles significantly affects their initial adsorption on biological membranes, and subsequent internalization of particles through endocytosis mechanisms largely depends on their size. For this reason, zeta potential and particle dimensions are closely related to the biological effects of nanoparticles and potential toxic responses [40,41].

Science has established that larger absolute values of zeta potential improve the stability of dispersions due to electrostatic repulsion, while decreasing zeta potential values weaken dispersion stability. That is, systems with larger absolute zeta values are more likely to remain stable due to electrostatic repulsion. Smaller zeta potential values allow van der Waals attraction to overcome repulsion, leading to particle aggregation [42].

Nevertheless, zeta potential should not be regarded as a single universal criterion of colloidal stability, as the behavior of a dispersion is also influenced by the composition and ionic strength of the medium, pH, particle concentration, and other physicochemical characteristics of the system [36,38].

#### 2.2.4. Biochemical Properties (Determination of Dry Matter Content, Determination of Soluble Solids, the pH, High Performance Liquid Chromatography (HPLC) Method for the Determination of Phenolic Compounds)

Determination of the *dry matter content* in berry pomace is a key physicochemical characterization parameter that allows for an accurate assessment of the raw material’s composition and its technological value. Dry matter refers to all the material remaining after water removal, including proteins, carbohydrates, fiber, phenolic compounds, and mineral substances. Knowing this parameter is essential for evaluating the pomace’s potential as a source of functional food ingredients, optimizing processing processes (e.g., drying or extraction), and predicting product stability and safety (as lower moisture reduces microbiological spoilage risk). In scientific research, the dry matter content is often determined thermogravimetrically—the sample is dried to a constant mass at a specific temperature, and the mass difference between the initial and dry state is used to calculate the moisture/dry matter ratio. An example of such methodology is normative procedures where samples are dried at 105 °C to constant mass, based on AOAC standard principles, to achieve reliable and comparable results in various plant-based products [43].

The determination of *soluble solids content* is an important indicator for assessing the chemical composition, technological properties, and quality of plant raw materials or their processing products. Soluble solids mainly include sugars (glucose, fructose, sucrose), organic acids, amino acids, soluble pectins, and mineral substances; thus, their amount is directly related to the nutritional value, taste, and processing suitability of the product. In the studies of berries and their pomace, this parameter allows for the assessment of the raw material’s maturity, juice extraction efficiency, and the concentration of biologically active compounds. Additionally, the content of soluble solids is often used to predict the progress and efficiency of product fermentation, concentration, or drying processes.

The amount of soluble solids is most commonly determined by the refractometric method, which is based on measuring the refractive index of light in a solution. A digital refractometer PR-32 (Atago Co., Ltd., Tokyo, Japan) was used for this study, allowing for quick and accurate determination of the amount of soluble solids, expressed in °Brix units [44,45]. This method is widely applied in food science due to its simplicity, the small sample requirement, and high measurement accuracy. The analysis of soluble solids provides important information about the quality of raw materials, enables optimization of technological processes, and allows comparison of the efficiency of different raw materials or processing methods [46].

Scientific research has determined that various factors, such as solution temperature and acidity, significantly influence the size, shape, and optical properties of nanoparticles (NP). Among these factors, the *pH* of the solution has a particularly significant impact on the morphology of silver nanoparticles (AgNP).

**Figure 5:**
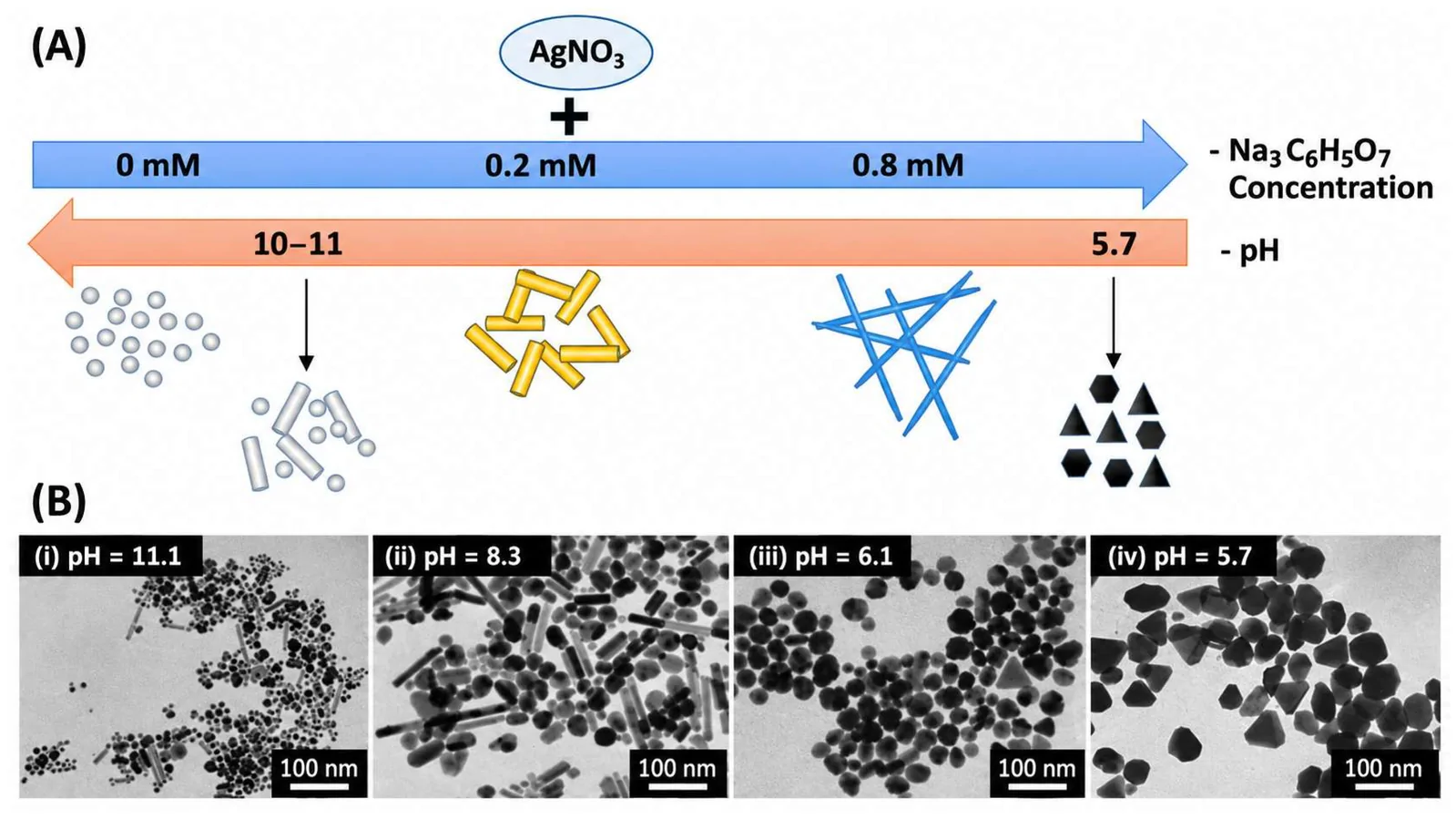
Synthesis of silver nanoparticles (AgNPs) in citrate medium: (**A**) influence of synthesis conditions on AgNPs morphology; (**B**) transmission electron microscopy images depicting AgNPs synthesized under different pH conditions: (**i**) pH 11.1; (**ii**) pH 8.3; (**iii**) pH 6.1 and (**iv**) pH 5.7, adapted from [32].

In the study conducted by Dong et al. (2009), it was shown that the shape of AgNP is strongly dependent on pH when citrate is used as a reducing and stabilizing agent (Figure 5A). The authors found that at high pH, a mixture of spherical and rod-shaped particles is formed, while at low pH, triangular and polygon-shaped nanoparticles dominate (Figure 5B) [32].

This phenomenon was confirmed in 2010 by Qin and co-authors, who found that increasing the medium’s pH and using ascorbate as a reducing agent and citrate as a stabilizer resulted in more spherical nanoparticles. The study also found that the size of the nanoparticles depends on the medium’s pH. As the pH increased, the reductive activity of citrate and ascorbic acid was enhanced, leading to the formation of smaller silver nanoparticles. This is associated with changes in the nucleation processes and their growth dynamics during synthesis [32].

The significance of *phenolic content* in berry pomace is an important indicator in food science, technology, and the study of bioactive substances, as phenolic compounds are the main antioxidants that contribute to:

- Antioxidant properties and health benefits: Phenolic compounds, such as flavonoids, phenolic acids, and anthocyanins, possess strong antioxidant properties; they neutralize free radicals and thus can reduce oxidative stress. These properties correlate with a lower risk of cardiovascular diseases, inflammatory processes, and certain chronic diseases [47].
- Source of valuable bioactive components: Berry pomace, which is produced as a by-product in juice production, often contains a much higher amount of phenolic compounds than the juice itself because many phenolics are concentrated in the skin and seeds. For example, the phenolic compound content in berry pomace can be 25–50 times higher than the phenolic content of the corresponding juice [48].
- Antioxidant activity indicators: Research shows that a higher phenol content in berry press residues often indicates greater antioxidant activity (DPPH and FRAP tests), making phenols a good indicator of the potential for functional ingredients [48].
- Possibility of application in the food, cosmetics, and pharmaceutical industries: Due to their biological activity, phenolic compounds from pomace can be used as natural antioxidants, food additives, or ingredients in nutraceutical and cosmeceutical products [49].

#### 2.2.5. Antioxidant Activity (Determination of Antioxidant Activity Using HPLC-ABTS and HPLC-FRAP Post-Column Assays)

Determining antioxidant activity allows for the assessment of the biological value of pomace and its potential use in the production of functional food ingredients, food additives, pharmaceutical, or cosmetic products. Antioxidant activity refers to the ability to neutralize free radicals and slow down oxidative processes that can damage biological molecules such as lipids, proteins, and DNA. In plant raw materials, including fruits, berries, and vegetables, the main antioxidants are phenolic compounds (flavonoids, phenolic acids, anthocyanins), vitamins (especially C and E), and carotenoids [50]. Antioxidant activity in extracts is usually determined by spectrophotometric methods: The DPPH test measures how the sample neutralizes DPPH radicals; the ABTS test evaluates the sample’s ability to inhibit ABTS radical absorption; the FRAP test determines the sample’s ability to reduce iron ions (Fe^3+^ → Fe^2+^) [51]. These methods allow for quantitative comparison of antioxidant activity between different types of extracts and establish a correlation with the amount of phenolic compounds [52].

#### 2.2.6. Antimicrobial Activity (The Agar Diffusion and Minimal Inhibitory Concentration (MIC) Tests Were Chosen to Evaluate Antibacterial Activity. The Antimicrobial Activity of Extracts Was Tested via an Agar Well Diffusion Assay)

Methods using various plant extracts are widely applied for the synthesis of silver nanoparticles (Ag NPs) to obtain nanostructures with different capping molecules and diverse morphologies. Scientific literature reveals that during plant-mediated Ag NP synthesis, bioactive molecules present in the extract act as stabilizing and capping layers, which significantly influence nanoparticle formation during their growth stage. The presence of the capping layer determines not only the morphology of Ag NP but also their size distribution. Moreover, the use of medicinal plant extracts in Ag NP synthesis is applied not only for controlling structural parameters such as size and shape but also to impart additional functional properties to the nanoparticles related to the antimicrobial activity present in plants [53].

The agar diffusion test (also known as the “disk diffusion” or “well diffusion” method) is a standard microbiological method used to evaluate the effect of antimicrobial substances on bacteria or fungi. The procedure is based on the diffusion of the antimicrobial substance through the agar medium, inhibiting the growth of microorganisms. The result is measured as the diameter of the zone around the disk or “well” where growth is inhibited. A larger inhibition zone generally indicates a stronger antimicrobial effect. The purpose of this test is to quickly compare the antimicrobial activity of various substances, extracts, or compounds and to assess antibiotic sensitivity [54].

The Minimum Inhibitory Concentration (MIC) is the lowest concentration of an antimicrobial substance that completely inhibits the growth of a microorganism in vitro. The MIC test is usually performed in liquid medium (in microplate or test tube format) or on an agar base. This method is used when it is necessary to accurately assess the activity of an antimicrobial compound, compare the effectiveness of different substances, and determine effective concentrations for antibiotics or natural extracts. MIC is most commonly determined using 96-well microtitration plates. Bacteria are inoculated into a liquid growth medium containing different concentrations of the tested antimicrobial agent. After incubation (16–20 h), bacterial growth is assessed and the MIC value is read. This method is applied only to aerobic bacteria and typically takes up to 3 days [55].

Both methods are often used together, especially when evaluating natural extracts, antibiotics, or new antimicrobial compounds [56].

## 3. Synthesis of Metal Nanoparticles

Nanoparticles can be produced using two main approaches: “top-down” or “bottom-up” methods. The “top-down” principle is based on breaking down larger materials into nanoscale particles using mechanical or physical processes, such as lithography, milling, laser ablation, or grinding. However, this method often requires a lot of energy and does not always allow precise control of particle properties, limiting its application in the production of high-quality nanoparticles.

Meanwhile, the “bottom-up” approach is based on the formation of nanoparticles from atoms or molecules using chemical or biological processes. This method allows for better control of the surface properties, shape, and size of nanoparticles. “Bottom-up” strategies include methods such as chemical reduction, sol–gel technology, and biological synthesis techniques [57].

### 3.1. Physical and Chemical Methods

During mechanical milling using high-energy ball mills, a fine material microstructure is formed. In the laser ablation process, by applying a laser to the target surface, ultra-pure nanoparticles can be obtained, and physical vapor deposition allows precise control of particle size and shape using complex vacuum technologies. Meanwhile, chemical reduction is characterized by simplicity, and the sol–gel method ensures material integrity and easy scaling of the process. Hydrothermal synthesis allows for the extraction of very pure and well-crystallized nanoparticles, and the co-deposition method helps maintain uniform composition, as all materials are deposited simultaneously [58,59].

Despite the ability to produce NPs with precisely controlled size and structure, traditional methods have significant drawbacks. Physical methods are energy-intensive, require complex equipment, and have low efficiency. Chemical synthesis often uses toxic substances that pose environmental and health risks, complicating product purification. Additionally, chemically obtained NP often have poor biological compatibility and degradability, limiting their application in biomedicine and environmental protection [60,61,62].

### 3.2. Biological Methods: Green Synthesis

Scientists have developed various nanoparticle synthesis methods that have a positive impact on the environment, as they are based on clean, non-toxic, and eco-friendly, green chemistry principles. These processes use living organisms, such as bacteria, fungi, and plants [63].

#### 3.2.1. Synthesis Based on Bacteria

Bacteria or bacterial extracts produce natural molecules (e.g., enzymes, peptides) that can reduce metal ions to nanoparticles and simultaneously stabilize them, preventing the particles from sticking to each other. The advantages of this method include rapid growth, simple genetic modification, a wide range of metabolic processes, the ability to conduct both intracellular and extracellular synthesis, and regulation of cell morphology, i.e., bacteria can change synthesis conditions (pH, temperature), thus affecting particle size and shape [64]. Using bacteria often results in a clean process without solvents, leading to potentially lower amounts of toxic residues [65].

However, this method also has drawbacks—some strains can be pathogenic, sterile conditions must be maintained, additional processing is required to separate nanomaterials, and there remains a risk of endotoxin contamination [64]. To extract nanoparticles, special steps may be required to separate them from bacterial cells and the filter, making the process more complex and labor-intensive [66]. Bacterial synthesis is sometimes inconsistent, so the properties of nanoparticles may vary in different batches [67].

#### 3.2.2. Synthesis Based on Fungi

Mushrooms (with their enzyme or extract mixtures) produce large amounts of proteins and other natural compounds that help convert metal ions into nanoparticles and stabilize them. The advantages of this method include intensive enzyme secretion, resistance to heavy metal exposure, ease of use, applicability for industrial-scale production, and the resulting nanomaterials often exhibit high stability. Speed and yield can be better than in the case of several bacteria, because fungi produce a larger quantity of bioactive molecules. Fungal extracts often form a stable, naturally protected nanoparticle dispersion due to their proteins and other biopolymers [64].

Cultivation of mushrooms and preparation of extracts can be slower and more complex than the simpler use of plant extracts [68]. Due to the complex composition of the mixture, it is sometimes difficult to know exactly which components are the most active, making it challenging to precisely control the structure of nanoparticles [64].

#### 3.2.3. Plant-Based Synthesis

Compared to other green nanomaterial (NM) synthesis methods, plant-mediated synthesis is considered the most efficient, as it allows for the production of large quantities of product. Such high NM yield is due to the good stability of the synthesized nanomaterials across various plant species [68].

In plant extract-mediated green synthesis of silver nanoparticles, silver nitrate (AgNO_3_) is commonly used as a precursor of Ag^+^ ions. The formation of AgNPs involves the reduction in Ag^+^ ions to metallic silver (Ag^0^), followed by nucleation, nanoparticle growth, and stabilization [69,70]. Plant extracts contain a complex mixture of phytochemicals that may act as reducing and stabilizing agents, including polyphenols, flavonoids, phenolic acids, tannins, terpenoids, polysaccharides, alkaloids, and other plant metabolites [69,70].

The principal reduction step can be represented in a simplified form as:

Ag^+^ + e^−^ → Ag^0^

During this process, certain plant-derived biomolecules act as electron donors. Phenolic compounds and flavonoids are particularly important because their hydroxyl (–OH) groups may participate in the reduction in Ag^+^ ions. For example, the catechol moiety of quercetin can donate electrons to Ag^+^ ions, facilitating their reduction to Ag^0^ [69]. More broadly, phytochemical functional groups, including hydroxyl, aldehyde, ketone, carboxyl, and amino groups, have been reported to be capable of participating in the reduction in Ag^+^ ions [70].

The role of plant-derived biomolecules is not limited to Ag^+^ reduction. Following the formation of Ag^0^ and subsequent nucleation and nanoparticle growth, some compounds present in the extract may adsorb onto the AgNP surface and act as capping or stabilizing agents. Functional groups such as hydroxyl (–OH), carbonyl (C=O), and carboxyl (–COOH) groups can interact with the nanoparticle surface, contributing to the formation of a stabilizing layer that limits particle aggregation and enhances colloidal stability [69].

However, plant extracts are complex multicomponent systems; therefore, the reduction in Ag^+^ cannot generally be attributed to a single compound or automatically to all members of a particular phytochemical class. Experimental studies investigating individual compounds identified in plant extracts have demonstrated that different phytochemicals may perform distinct functions during AgNP formation. For example, Pradeep et al. showed that phenolic acids and flavonoids present in Hypericum perforatum extract were involved in Ag^+^ reduction, whereas xanthones and phloroglucinols primarily acted as capping agents, and naphthodianthrones participated in both processes [71]. These findings indicate that plant-mediated AgNP synthesis results from the combined action of several phytochemicals and their functional groups, with their respective contributions depending on the specific phytochemical composition of the plant material [69,71].

Plant extracts are easily accessible and inexpensive, making synthesis simpler in many cases than with microorganisms [65]. The process often occurs at room temperature without toxic substances, making it safe and less energy intensive. The plant method poses no biological hazard associated with the handling of live microbial cultures, making it suitable for synthesis without the cultivation requirements associated with microorganism-based methods [69].

As a negative aspect, the composition of plant extracts can vary depending on the type of plant, season, and preparation method, making the control of nanoparticle size and shape more challenging [65]. Sometimes, plant extracts produce particles of various shapes and sizes, which limits their application in areas where much more uniform particles are needed [68].

Reviews of plant synthesis emphasize that it is an environmentally friendly and economical alternative, but reproducibility and size control remain challenges [72].

In summary, based on the scientific literature describing biological synthesis methods, several key advantages of these approaches can be highlighted. Biological methods avoid the use of toxic chemical reducing agents and solvents, making the synthesis process more environmentally friendly and safer [65]. They can also be performed at lower temperatures and pressures than conventional chemical methods [73]. Furthermore, the resulting nanoparticles are often biocompatible, which is particularly important for medical and environmental applications [74].

**Figure 6:**
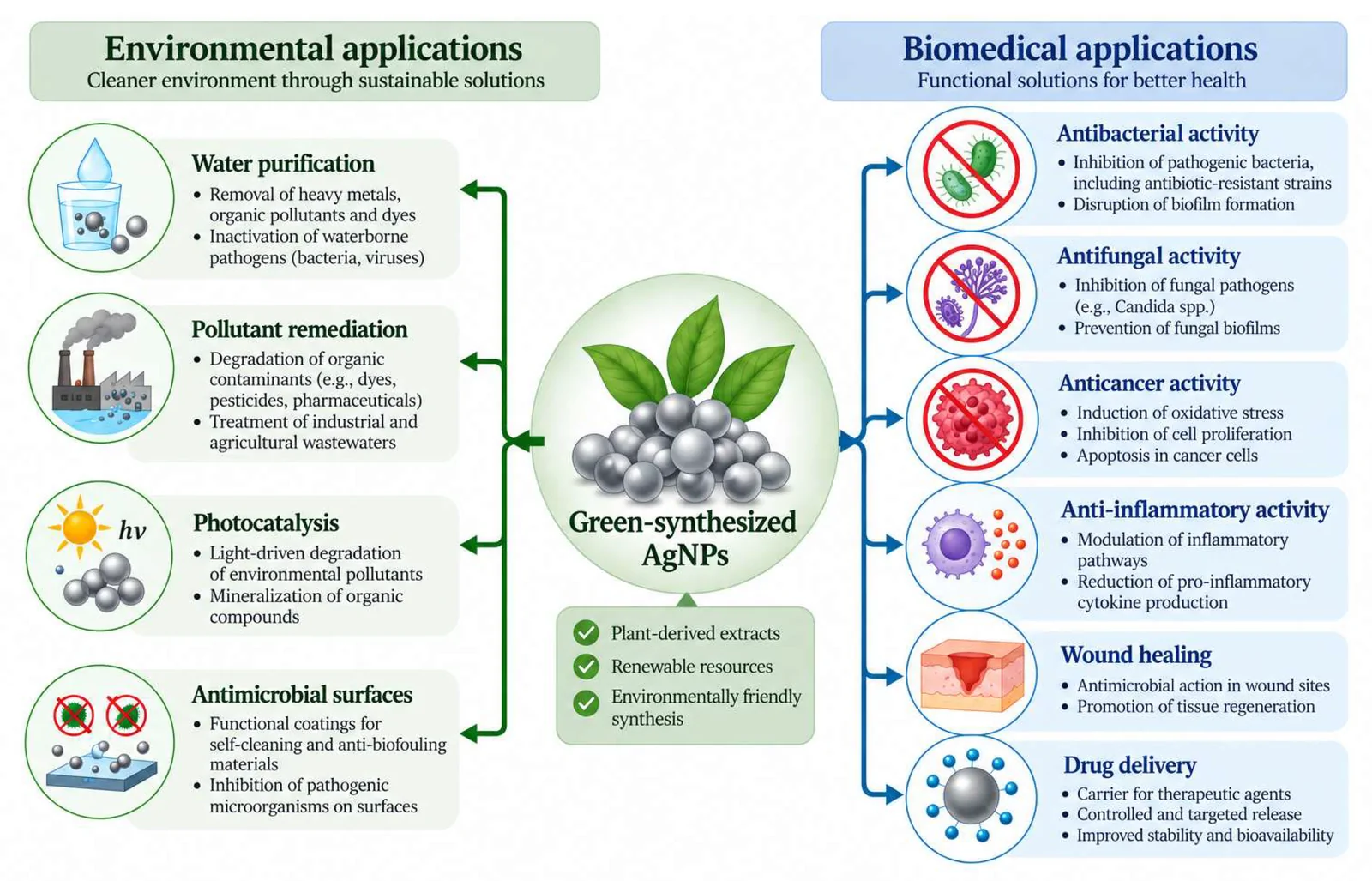
Schematic overview of the main environmental and biomedical applications of green-synthesized silver nanoparticles (AgNPs), based on the literature [63,76,77].

However, several challenges associated with biological synthesis methods should also be considered. Compared with chemical methods, precise control over nanoparticle size, shape, and uniformity can be more difficult to achieve [68]. Production yields and batch-to-batch consistency may also vary [67]. In addition, some biological synthesis methods remain difficult to scale up to industrial production because process standardization and large-scale production have not yet been fully established [68]. Thus, various methods are available for NP synthesis, and the selection of the most appropriate approach depends on the intended application and the required nanoparticle properties, including particle size, shape, and composition [75]. More comprehensive toxicological and safety studies are needed to clarify how biological nanoparticles affect humans and the environment [65] (Figure 6).

## 4. Chemical Composition and Potential Uses of Fruits, Berries and Vegetables Processing By-Products

### 4.1. Key Indicators of Bioactive Materials for Secondary Recycling Materials

By-products generated during the processing of fruits, berries, and vegetables, such as peels, seeds, and pomace (mass residues), are rich in compounds of biological and nutritional value. They contain proteins, fibers, polysaccharides (e.g., pectin).

Vitamins, minerals, phytochemical antioxidants, polyphenols, carotenoids, flavonoids, lipids, and plant oils have great potential to be used as functional food substances and additives for health products, and can be a source of bioactive components for the food, cosmetics, or pharmaceutical industry, for the formation of biodegradable protective layers and films (as an alternative to plastic), and components for active packaging, biofuels, biogas, or biofertilizers [63]. Many plant polysaccharides, such as pectin, cellulose, and hemicellulose, are suitable for producing biopolymers and fibers [78]. Phenolic compounds and flavonoids are strong antioxidants used in the food, health products, and cosmetics industries [63]. Carotenoids, vitamins, and organic acids are functional components suitable for creating food supplements and natural colorants [79]. Plant proteins and lipids can be extracted and used in other production processes [80].

### 4.2. Chemical Composition and Antibacterial Activity of Fruit, Berry, and Vegetable By-Products

The composition of extracts from chemical by-products of fruits, berries, and vegetables describing antibacterial activity is presented in Table 2.

### 4.3. Chemical Composition of Fruit, Berry, and Vegetable By-Products Extracts Characterizing Antioxidant Activity

The composition of chemical extracts from by-products of fruits, berries, and vegetables, describing antioxidant activity, is presented in Table 3.

### 4.4. The Content of Bioactive Compounds in Fruit, Berry, and Vegetable Pomace

Fruit juice production is one of the largest sectors of the agricultural industry, often using apples, sea buckthorn, carrots, cherries, cranberries, oranges, peaches and other fruits and berries, and even vegetables. This production generates a large amount of waste, including peels, seeds, pulp, press residues, stems, and pits. These wastes are rich in bioactive compounds with antioxidant, anti-inflammatory, and anticancer effects. Therefore, their utilization and value enhancement are extremely important [105]. This section reviews studies conducted by scientists examining the content of bioactive compounds in fruit, berry, and vegetable pomace (production by-products), with the main components and key results presented in Table 4.

## 5. Release of AgNPs from Film to Food

The identification and characterization of nanomaterials in the food chain is essential to assess potential risks to consumer safety, as these particles can migrate from packaging into food. This requires the use of specialized methods for the analysis and characterization of nanomaterials: microscopy, quantification, and spectroscopy [145]. Literature data indicate that controlling the size of silver nanoparticles and the precise composition and properties of other biopolymer materials can effectively control their migration into food, thereby reducing the potential exposure of consumers to nanoparticles. Literature data show that controlling the size of silver nanoparticles and the precise composition and properties of other biopolymeric materials can effectively control their migration into food, thereby reducing potential consumer exposure to nanoparticles. Higher amounts of silver nanoparticles in the human body can be dangerous to many systems, such as the skin, eyes, kidneys, respiratory tract, liver and biliary tract, and immune system [146].

## 6. Cytotoxicity of AgNPs

The cytotoxic effects of silver nanoparticles (AgNPs) are considered to be potentially beneficial in modern nanomedicine, especially in the development of new strategies for anticancer treatment and the fight against antibiotic-resistant bacterial infections and other diseases. In this context, targeted therapy systems are actively being investigated to selectively target pathological cells and avoid damage to healthy tissues. This opens up the possibility of controlled synthesis of both low-toxicity and increased-biological-activity AgNPs, depending on their intended application [146]. Studies show that the cytotoxicity of AgNPs increases significantly as the size of the nanoparticles decreases. Literature data show that not only the size, but also the shape and surface charge of nanoparticles have a significant impact on their toxicity. Studies investigating the relationship between surface charge and toxicity have shown that increasing negative surface charge reduces the toxicity of nanoparticles, while positively charged nanoparticles have a higher bactericidal activity than negatively charged particles [147].

However, the cytotoxicity of AgNPs is not associated solely with their desirable antibacterial or anticancer effects. When interacting with biological systems, AgNPs may also induce adverse toxic effects. AgNP toxicity has been associated with several interrelated mechanisms, including the generation of reactive oxygen species (ROS) and oxidative stress, mitochondrial dysfunction, DNA damage, and the activation of apoptotic processes. The magnitude of these toxic effects depends on particle size, shape, surface properties, concentration, and exposure conditions, and in vivo studies have also demonstrated potential effects of AgNPs on cells, tissues, and organs [146]. Therefore, the safety assessment of AgNPs should not be limited to the determination of cytotoxicity in individual cell cultures but should also include a broader evaluation of their effects on biological systems.

The potential environmental impact of AgNPs, particularly on aquatic ecosystems, is equally important. The ecotoxicity of biosynthesized AgNPs was directly demonstrated by Khoshnamvand et al., who investigated AgNPs synthesized using *Alcea rosea* leaf extract at three trophic levels of a freshwater food chain: the microalga *Chlorella vulgaris*, the zooplankton *Daphnia magna*, and the fish *Danio rerio*. The plant-derived capping material associated with the AgNPs did not exert toxic effects on the tested organisms, whereas the biosynthesized AgNPs were toxic to all three trophic levels. Although Ag^+^ ions were more toxic than the AgNPs, their release from the stable nanoparticles was low; therefore, the authors attributed the observed effects predominantly to the nanoparticles themselves (148).

These findings also demonstrate that the use of green or biological synthesis methods does not inherently guarantee that the resulting AgNPs are non-toxic. Green synthesis can reduce the use of hazardous chemical reducing and stabilizing agents, while plant-derived or other biological compounds may serve as reducing and stabilizing agents. Nevertheless, the biological effects of the resulting AgNPs depend on their physicochemical characteristics, surface properties, concentration, and interactions with the specific biological or environmental system. Therefore, green synthesis should be regarded as a more sustainable approach to nanoparticle production rather than as evidence of the toxicological safety of the resulting nanoparticles [146,148].

Another important consideration is the duration of exposure. Although the toxicology of AgNPs has been extensively investigated, knowledge regarding their long-term effects remains limited. Noga et al. emphasized that, with increasing exposure of humans, animals, and the environment to nanoparticles, important knowledge gaps remain regarding both the short- and long-term toxicological risk of AgNPs. Therefore, it would be inaccurate to state that long-term toxicity studies of AgNPs are entirely lacking; rather, the available data on long-term and chronic effects remain limited and are insufficient for a comprehensive assessment of long-term risks, particularly under different realistic biological and environmental exposure conditions [148].

Nevertheless, the potential toxicity of AgNPs does not in itself preclude their practical application. Owing to their strong antimicrobial properties, AgNPs remain promising components of active food packaging systems. Incorporation of AgNPs into biopolymeric matrices can provide packaging materials with antimicrobial activity and thereby contribute to extending the shelf life of food products. Moreover, incorporation of AgNPs into biopolymers may improve certain physical and mechanical properties of the resulting materials [149].

Therefore, the application of AgNPs should not be based on the assumption that these nanoparticles are non-toxic, but rather on an assessment of the balance between their functional benefits and potential risks. In food-packaging applications, particular attention should be paid to the possible migration of silver nanoparticles or other silver species from the polymeric matrix into food and the resulting consumer exposure. Studies have demonstrated that the migration of silver from nanocomposite packaging may depend on several factors, including the packaging material, the nature of the food or food simulant, contact time, and temperature. Moreover, the available results concerning AgNP migration are not fully consistent, highlighting the need for further investigation into nanoparticle migration and its potential toxicological implications [145].

Thus, the promising application of AgNPs, particularly in active biopolymer-based food packaging, should be accompanied by controlled nanoparticle use and safety assessment of the specific system. The development of such materials should focus on optimizing AgNP concentration, physicochemical and surface properties, and interactions with the biopolymeric matrix, while simultaneously evaluating antimicrobial efficacy, potential migration, and toxicological and environmental risks. Such an approach may allow the functional benefits of AgNPs to be retained while minimizing unnecessary consumer and environmental exposure [145,149].

## 7. Conclusions and Future Perspectives

Silver nanoparticles (AgNPs) remain one of the most promising functional materials for active food packaging due to their exceptional antimicrobial properties, high surface reactivity, and ability to effectively inhibit the growth of pathogenic microorganisms. In recent years, there has been a growing interest in “green” synthesis methods that use bioactive compounds such as polyphenols, flavonoids, and organic acids from fruits, berries, and vegetable extracts to synthesize AgNPs. These natural extracts act as reducing and stabilizing agents, allowing the production of biocompatible, less toxic nanoparticles and reducing the need for synthetic chemicals and environmental pollution.

AgNPs obtained in this way are successfully incorporated into biopolymer matrices such as chitosan, starch, alginates, or polylactic acid, forming nanocomposite films and coatings. These multifunctional systems exhibit improved mechanical properties, increased barrier resistance to water vapor and oxygen, better thermal stability, and enhanced antimicrobial activity. Importantly, the biopolymer matrix also allows for the control of the release of silver ions and nanoparticles, thereby reducing potential cytotoxic effects and ensuring safer food contact.

Studies have shown that such green AgNP-based biopackages are effective in extending the shelf life of various perishable foods, including fresh fruits, berries, vegetables, dairy products, fish, and meat. Due to these properties, they are considered a promising approach for sustainable food packaging technology that meets both food safety and environmental requirements.

## Figures and Tables

**Table 1:** Relationship between AgNP physical properties and color indices.

AgNP Size/Morphology	UV–Vis SPR Absorption Maximum (_λmax_)	Color in Colloidal Solution	Color Indices (Examples)
~10–20 nm, spherical	~390–420 nm	Yellow–light brown	✔ RGB (yellow) ✔ CIE L*a*b* b* high positive (yellow)
~20–40 nm	~420–450 nm	Darker brown	✔ RGB (red/yellow) ✔ CIE L*a*b* b* positive
≥50 nm/anisotropic	~450–500 nm or higher	Brown–dark brown/orange	✔ CIE L*a*b* a*, b* vary according to shape

**Table 2:** The composition of extracts from chemical by-products of fruits, berries, and vegetables describing antibacterial activity.

Statement	Chemical Components/Properties	Antibacterial Activity Indicators	Links
1. The phenolic compound content > ~40 mg GAE/g is associated with significant antibacterial activity in various F&V by-product extracts.	High TPC is a complex of phenols, flavonoids, phenolic acids from berry and fruit waste	Weak to moderate antibacterial activity in vitro; the antibacterial effect of such extracts often depends on the concentration and type of bacteria.	[81,82,83,84]
2. Extracts of plant by-products with TPC ≥ ~100 mg GAE/g show stronger antibacterial effects.	For example, certain extracts of black berries, pomegranate peels, or grape seeds have a very high polyphenol content.	Studies have shown that higher TPC is associated with broad antibacterial effects against Gram-positive and Gram-negative bacteria. Extracts with such TPC show inhibition of S. aureus, E. coli, etc. Medium strength antibacterial activity in vitro, often significantly lower MIC values (lower extract quantity required for bacterial inhibition) than extracts with lower TPC.	[81,82,83,85,86]
3. TPC between ~150–250+ mg GAE/g can indicate very strong antibacterial activity, often exceeding 500 µg/mL MIC.	The TPC of Mediterranean F&V by-product extracts ranged from ~44 to >250 mg GAE/g, and stronger antibacterial effects were associated with higher TPC values.	In studies where higher TPC values were observed, more pronounced growth inhibition of Staphylococcus, Listeria, Bacillus, and other bacteria was identified. The highest concentrations of phenolic compounds are often associated with very strong antibacterial effects against various bacterial strains in vitro, often indicating a stronger effect at lower extract concentrations.	[81,82,83,87]

**Table 3:** Chemical composition of fruit, berry, and vegetable by-product extracts characterizing antioxidant activity.

Bioactive Compound	Key Indicators/Features	Usage	Links
Phenolic compounds (polyphenols)	- Strong antioxidant activity - Chemical reduction capacity for metal ions - Functional groups (OH, COH)	- Reduce Ag^+^ to AgNPs and stabilizes nanoparticles - Increase the oxidative stability of biopolymers - Participate in cross-linking in films	[88,89,90,91]
Flavonoids	- Antioxidant activity - Chelating properties for metals - Help manage nanoparticle morphology	- Participate in the reduction and stabilization of AgNP synthesis - Improve the monodispersity of the nanoparticles - Improve the bioactivity of biopolymer films	[89,90,92]
Carotenoids	Pigmentation, antioxidant stability, lipophilic properties	Act as lipid-phase antioxidants and enhance the oxidative stability of biopolymer matrices and nanostructured systems	[93,94,95]
Plant proteins	Reducing and encapsulating properties - Form natural stabilizers	Can form biopolymeric matrices, stabilize silver nanoparticle (AgNP) surfaces, improve nanoparticle dispersion, and enhance biocompatibility	[96,97,98,99,100]
Polysaccharides (pectin, cellulose, hemicellulose)	- Large molecular mass - Abundance of functional groups (OH, COOH) - Flowering ability	- Act as the primary matrix for biopolymers, forming films, hydrogels, or nanocomposites - Stabilize nanoparticles via hydrogen bonding, van der Waals interactions, and electrostatic interactions, preventing aggregation - Improve the mechanical properties and colloidal stability of nanostructures	[100,101,102]
Organic acids	Complexation of metal ions, pH regulation	Help to form monodisperse AgNPs - Regulate the physical properties of biopolymers	[103,104]

**Table 4:** Bioactive compounds in fruit, berry, and vegetable production by-products, their potential for use.

Raw Material	Scientific Name	Main Pomace Components	Key Bioactive Compounds	Application Potential	Link
Apple	*Malus domestica*	Peel, seeds, pulp	Pectin, Polyphenols, flavonoids	Biopolymer films, AgNP reduction	[106]
Pear	*Pyrus communis*	Peel, pulp	Polyphenols	Antioxidant biopolymers	[107]
Grape	*Vitis vinifera*	Skins, seeds	Resveratrol, tannins	Reinforcement, antimicrobial films	[108]
Orange	*Citrus sinensis*	Peel, albedo	Pectin, hesperidin	Film-forming polymers	[109]
Lemon	*Citrus limon*	Peel	Flavonoids, citric acid	AgNP green synthesis	[110]
Grapefruit	*Citrus paradisi*	Peel	Pectin, naringin	Active packaging	[111,112]
Mandarin	*Citrus reticulata*	Peel	Polymethoxyflavones	Antimicrobial films	[113]
Pomegranate	*Punica granatum*	Peel, seed coats	Ellagitannins	AgNP stabilization	[114]
Mango	*Mangifera indica*	Peel, kernel	Phenolics, starch	Biopolymer matrices	[115]
Pineapple	*Ananas comosus*	Peel, fiber	Cellulose	Biocomposite reinforcement	[116]
Plum	*Prunus domestica*	Peel, pulp	Anthocyanins	Antioxidant films	[117,118]
Apricot	*Prunus armeniaca*	Peel, pulp	Carotenoids	Active packaging	[119]
Peach	*Prunus persica*	Peel	Polyphenols	Functional polymers	[120]
Blackcurrant	*Ribes nigrum*	Skins, seeds	Anthocyanins, chlorogenic acid and caffeic acid	AgNP reduction	[121,122]
Red currant	*Ribes rubrum*	Skins	Phenolic acids	Antioxidant films	[123]
Blueberry	*Vaccinium corymbosum*	Skins	Anthocyanins	Biopolymer additives	[124,125]
Bilberry	*Vaccinium myrtillus*	Skins	Polyphenols	Antimicrobial systems	[125]
Cranberry	*Vaccinium macrocarpon*	Skins	Proanthocyanidins, Procyanidins	Active packaging	[126,127]
Raspberry	*Rubus idaeus*	Seeds	Ellagic acid	Film reinforcement	[128]
Blackberry	*Rubus fruticosus*	Seeds	Polyphenols	Antimicrobial biopolymers	[129]
Strawberry	*Fragaria × ananassa*	Pulp residues	Phenolics	Edible films	[130]
Sea buckthorn	*Hippophae rhamnoides*	Peel, seeds	Flavonoids, lipids	AgNP synthesis	[131]
Guelder rose berries (Viburnum)	*Viburnum opulus*	Skins, seeds, pulp residues	Phenolic acids, flavonoids, iridoids	Strong reducing and stabilizing agent for green synthesis of AgNPs; antioxidant and antimicrobial biopolymer additives	[132,133]
Aronia	*Aronia melanocarpa*	Skins	High polyphenols	Strong reducing agent	[134]
Rosehip	*Rosa canina*	Peel, seeds	Ascorbic acid	Antioxidant polymers	[135,136]
Carrot	*Daucus carota*	Fiber pulp	Cellulose, carotenoids	Biopolymer reinforcement	[137,138]
Beetroot	*Beta vulgaris*	Pulp	Betalains	Functional films	[139]
Tomato	*Solanum lycopersicum*	Skins, seeds	Lycopene, β-carotene, lutein	Antioxidant packaging	[140]
Pepper	*Capsicum annuum*	Seeds, skins	Capsaicinoids	Antimicrobial packaging	[141]
Cucumber	*Cucumis sativus*	Peel	Phenolics	Edible coatings	[142]
Pumpkin	*Cucurbita* spp.	Pulp residues	Polysaccharides	Film-forming polymers	[143]
Zucchini	*Cucurbita pepo*	Pulp	Fiber	Biopolymer matrices	[144]

## Data Availability

No new data were created or analyzed in this study. Data sharing is not applicable to this article.
